# Tissue Effect on Genetic Control of Transcript Isoform Variation

**DOI:** 10.1371/journal.pgen.1000608

**Published:** 2009-08-14

**Authors:** Tony Kwan, Elin Grundberg, Vonda Koka, Bing Ge, Kevin C. L. Lam, Christel Dias, Andreas Kindmark, Hans Mallmin, Östen Ljunggren, Fernando Rivadeneira, Karol Estrada, Joyce B. van Meurs, Andre Uitterlinden, Magnus Karlsson, Claes Ohlsson, Dan Mellström, Olle Nilsson, Tomi Pastinen, Jacek Majewski

**Affiliations:** 1 Department of Human Genetics, McGill University, Montréal, Canada; 2 McGill University and Genome Québec Innovation Centre, Montréal, Canada; 3 Department of Medical Sciences, Uppsala University, Uppsala, Sweden; 4 Department of Surgical Sciences, Uppsala University, Uppsala, Sweden; 5 Department of Internal Medicine, Erasmus MC, Rotterdam, The Netherlands; 6 Department of Epidemiology, Erasmus MC, Rotterdam, The Netherlands; 7 Clinical and Molecular Osteoporosis Research Unit, Department of Clinical Science, Lund University and Department of Orthopaedics, Malmö University Hospital, Malmö, Sweden; 8 Center for Bone Research at the Sahlgrenska Academy, Department of Internal Medicine, Göteborg University, Gothenburg, Sweden; The University of Queensland, Australia

## Abstract

Current genome-wide association studies (GWAS) are moving towards the use of large cohorts of primary cell lines to study a disease of interest and to assign biological relevance to the genetic signals identified. Here, we use a panel of human osteoblasts (HObs) to carry out a transcriptomic survey, similar to recent studies in lymphoblastoid cell lines (LCLs). The distinct nature of HObs and LCLs is reflected by the preferential grouping of cell type–specific genes within biologically and functionally relevant pathways unique to each tissue type. We performed *cis*-association analysis with SNP genotypes to identify genetic variations of transcript isoforms, and our analysis indicates that differential expression of transcript isoforms in HObs is also partly controlled by *cis*-regulatory genetic variants. These isoforms are regulated by genetic variants in both a tissue-specific and tissue-independent fashion, and these associations have been confirmed by RT–PCR validation. Our study suggests that multiple transcript isoforms are often present in both tissues and that genetic control may affect the relative expression of one isoform to another, rather than having an all-or-none effect. Examination of the top SNPs from a GWAS of bone mineral density show overlap with probeset associations observed in this study. The top hit corresponding to the *FAM118A* gene was tested for association studies in two additional clinical studies, revealing a novel transcript isoform variant. Our approach to examining transcriptome variation in multiple tissue types is useful for detecting the proportion of genetic variation common to different cell types and for the identification of cell-specific isoform variants that may be functionally relevant, an important follow-up step for GWAS.

## Author Summary

The transcriptome of any given cell type is a complex program of controlled gene expression underlying its biological function. An additional layer of molecular complexity involving individual genetic variation can modulate the transcriptome within the same tissue type, conferring potential phenotypic differences between individuals at the cellular level. This study highlights common and unique aspects of the transcriptome between the well-characterized lymphoblastoid cell lines from the International HapMap Project and those of a cultured primary cell type, human osteoblasts. We observe that inter-individual genetic variation can regulate transcript isoform expression in tissue-specific and tissue-independent manners, indicating that genetic differences among individuals can alter the transcriptome in one or more tissues, ultimately leading to altered biological functions within the lymphoblasts and/or osteoblasts. Pursuant to this, genome wide association studies on bone mineral density (BMD) have identified a number of significant loci and polymorphisms highly linked to the BMD quantitative phenotype. A small proportion of these polymorphisms overlap with our highly significant SNPs regulating the osteoblast transcriptome, revealing a potential molecular basis for this phenotype at the transcriptional level. This study highlights the importance of examining the differing transcriptomes and *cis*-regulatory mechanisms governing the biological and functional roles of varied tissue types.

## Introduction

Human genetic variation plays a key role in shaping phenotypic diversity amongst individuals, where the underlying mechanisms may include polymorphisms that alter protein coding sequences or changes in regulatory sequences that affect the function of a gene or related gene networks. These alterations can affect heritable quantitative traits as well as susceptibility to common complex diseases [1]. Many early genome-wide association studies (GWAS) linking gene expression to genetic variation utilized lymphoblastoid cell lines (LCLs) and conclusively demonstrated the widespread impact of standing variation in governing-gene expression and population variability on expression traits [2],[3],[4]. These studies benefitted from the dense-genotyping information that is publicly available for the HapMap LCL samples [5]. Recently, using a subset (HapMap CEU) of these same LCLs, we demonstrated that genetic variants can be associated with expression changes at the exonic level and lead to alternatively spliced variants [6]. In a broader context, the expression of different transcript isoforms within individuals is partly under genetic control. Such isoform differences encompass inclusion or exclusion of cassette exons which may encode for important functional domains or structural motifs, or the alterations may be regulatory in nature with changes in the 5′ or 3′ untranslated regions leading to differences in translation initiation, mRNA stability, or mRNA localization.

Although the use of LCLs has generated tremendous amounts of useful information for genetic association and population genomics studies, this cell type does pose certain limitations. LCLs are immortalized cell lines and therefore may include potential artificial phenotypic and epigenetic alterations induced by immortalization and prolonged cell culture [7],[8],[9]. Even when LCLs are derived from a disease cohort of interest [10], they may not be truly representative of the regulatory landscape in the affected tissue of interest. Despite some of the caveats of LCLs, they have proved to be a valuable model system to establish patterns of heritable gene expression. Recently, the focus has expanded to examine genetic variation in other tissue types that may be more relevant to the disease or phenotype being studied, although large cohorts of all of these cell types are not always readily available. For example, genetic control of splicing in humans was examined using cortical brain tissue samples and peripheral blood mononucleated cell (PBMC) samples [11] but so far no studies on primary cultured cells have been performed.

In this study, we use cultured human osteoblasts (HObs), which are key players in the bone remodeling process including bone resorption and producton of bone matrix [12]. Osteoblasts are relevant to the study of osteoporosis, a skeletal disorder characterized by imbalances in the remodeling process, leading to compromised or weakened bone strength. Osteoblasts can be extracted from bone fragments, which are readily available from institutions performing routine hip replacement surgeries, making them a practical and useful resource for studying bone-related functions [13],[14],[15]. Using a similar strategy employed for the LCLs [6], we use the Affymetrix Human Exon Array for a population genomic examination of the human osteoblast transcriptome. Genotype information for our HOb panel was obtained using the Illumina Hap550K chip and used in a genome-wide association analysis with the exon expression data to identify transcript isoform variations showing genetic association to SNPs, and relate these isoform variants to potentially important biological roles in the bone remodeling process. This data was also compared with our previous results for the LCLs in order to detect common transcript isoform differences as well as tissue-specific variations. A similar approach was recently used for a comparison of brain and PBMCs [11] and the findings in that study will be discussed in the context of our results. Finally, our results were compared to the top SNP associations identified from a GWAS of bone mineral density [16],[17], which suggests that a significant proportion of hits may be involved in expression of alternative splice isoforms. We selected one example (*FAM118A*) from the overlapping hits for follow-up fine-mapping studies, which revealed a novel transcript isoform with potential functional importance in bone biology.

## Results

### Quality control of microarray data and population stratification

We investigated genetic variation on exon expression levels in a panel of 58 unrelated human osteoblast (HOb) samples of Swedish origin using the Affymetrix Human Exon 1.0 ST array (gender and year of birth detailed in Table S1). Expression values at the exon and gene levels were generated using the Robust Multichip Average (RMA) algorithm [18],[19]. Principal component analysis (PCA) was performed on the exon and gene level expression scores and did not identify any outliers for exclusion from the analysis (Figure S1). Genetic stratification was analyzed by EIGENSTRAT [20] and no indication of major population structure was seen in the osteoblast samples (P = 0.172446, Tracy-Widom test for first Eigen axis).

### Overall gene expression in osteoblasts and lymphoblasts

A mean expression score across 17793 genes was calculated and used as an arbitrary cutoff for presence/absence calls in a particular cell type. For HObs and LCLs, mean RMA expression scores of 6.193 and 6.196 were calculated, corresponding to 9227 (51.9%) and 9297 (52.3%) genes expressed, respectively in the two tissues. Although this mean expression value cutoff is conservative and may miss genes that are expressed at very low levels or just above array background, this cutoff was chosen so that we could compare a highly confident set of expressed genes from the two cell lines. From these two lists, 7733 (43.5% of the total) genes were expressed in common between the two cell types. These results are in line with a recent study using a similar set of samples on the Affymetrix Human Genome U133 Plus 2.0 Array [21].

We performed a network analysis using the Ingenuity Pathways Analysis (IPA) system on the sets of genes expressed uniquely in each cell type. The most significant or overrepresented networks corresponded to biological function(s) normally associated with that particular tissue (Table S4). The Wnt/β-catenin signaling, BMP (bone morphogenetic protein) signaling and IGF-1 signaling pathways were among the most significant HOb networks; these are known to be important in the bone remodeling process [22],[23],[24]. Conversely for the LCL-specific genes, the top canonical pathways included B-cell receptor signaling, NF-kB signaling, and natural killer cell signaling which are involved in the immune response system and are typically functions associated with lymphocytic cell types.

We also performed a network analysis for the set of 7733 shared HOb and LCL expressed genes (Table S3), and identified general cellular functions required for cell survival such as protein ubiquitination, glucocorticoid receptor signaling, PI3/AKT signaling, oxidative phosphorylation, and purine/pyrimidine metabolism. When this list is further subdivided into subsets of genes that are upregulated in either HObs or LCLs (>3×fold change mean expression) (Table S2), we again observe enrichment of biological networks that are intuitively predicted to be associated with that particular cell type (Table S5). Genes that are upregulated in HObs (n = 233) are preferentially found in integrin signaling, actin cytoskeletal signaling, and IGF-1 signaling pathways. Conversely, LCL-upregulated genes (n = 493) are found within antigen presentation, NF-kB signaling, and interferon signaling pathways. These results indicate that overall, there is a large overlap in genes expressed in the two cell types, as well as the associated functional pathways. However, by concentrating on the much smaller subset of cell type-specific genes, the networks analysis preferentially identifies biologically and functionally relevant pathways that are unique to both osteoblasts and lymphoblasts.

### Comparison of effects of common genetic variation on transcript isoforms between tissues

Before carrying out association analyses of meta-probeset-SNP and probeset-SNP associations, we note that there will be a decrease in the significance levels from the linear regression statistic for the HOb samples as compared to our previous work on LCLs. This is due to the fact that although similar sample sizes in LCLs and HObs (57 versus 58) were used, the expression values for LCLs are derived from the mean of three replicate samples whereas only a single HOb replicate is used. The increased statistical power generated from the use of triplicate samples will be reflected in lower variance between different samples and accordingly, a more significant P-value for true associations. This will be an important consideration when comparing associations between the two sample sets and looking at overlapping or unique genetic associations. Gender, age, batch effects, and the first eigen vector (from Eigenstrat analysis) were used as covariates in the regression analysis to account for any possible stratification.

We first examined the top 100 associations at the gene level in HObs and looked at the overlap of these genetic associations in LCLs, with the criteria that the gene must be expressed in both tissue types. Out of the top 100 unique genes and the top SNP association for each gene (P-values range from 7.298e-11 to 1.327e-04) in osteoblasts, we observed a 21% overlap in associations in lymphoblasts (nominal P-value<0.05). Multiple correction was also performed on the corresponding P-values for the same gene-SNP in lymphoblasts; 18% and 13% of associations were significant at 0.05 and 0.01 FDR levels, respectively. FDR was performed in consecutive 100 gene blocks in decreasing order of significance in HObs, with a resultant decreasing number of significant associations at both the 0.05 and 0.01 FDR levels in lymphoblasts (Figure S2A). This indicates an enrichment of common top associations between HObs and LCLs as well as a non-random nature of these top P-values. Conversely, when the top 100 gene-SNP associations in LCLs (P-values range from 3.347e-22 to 5.48e-06) were compared to HObs at a P<0.05 cutoff, 47% of associations were shared between the two cell types, and 37% and 23% were significant at 0.05 and 0.01 FDR levels, respectively, along with a similar decrease in shared associations for decreasing levels of significance in LCLs (Figure S2B).

A similar analysis was performed at the exon level, with the one major criterion to enrich for alternative splicing (AS) changes rather than whole gene expression changes. We limited ourselves to transcripts where the corresponding transcript (meta-probeset) did not show association to the same SNP showing association with the probeset. The top 100 probesets and the most significant SNP association for each probeset (P-values range from 4.93e-14 to 1.338e-5) in HObs showed 52% overlap of associations in LCLs (P<0.05) and 46% and 40% significant associations at 0.05 and 0.01 FDR levels, respectively. Conversely, the top 100 probesets in LCLs (P-values range = 6.247e-21 to 2.776e-7) showed 60% common associations in HObs (P<0.05) and 53% and 37% significant associations at 0.05 and 0.01 FDR levels, respectively. Similar to the analysis for the meta-probesets, FDR analysis on blocks of decreasing P-value significance for probesets in one tissue show a corresponding decreasing enrichment of significant associations in the other tissue (Figure S2C, S2D).

Clearly, for the top associations there are shared hits between HObs and LCLs at both the probeset and meta-probeset levels, suggesting that there is a common genetic control of transcript isoform expression that is independent of the types of tissues examined in this study.

In our previous study, we validated 25 transcript isoforms showing strong genetic association to SNPs in LCLs (P = 2.81e-37 to 3.53e-08) [6], and we sought to determine the level of replication in osteoblasts (Table 1). Validation was carried out by standard and quantitative real time RT-PCR on a panel of individuals with one or the other allele of the associated SNP in question. For two of the genes (*TAP2* and *C17orf57*), we were unable to detect any PCR product in HObs. Of the remaining 23 genes tested, 18 (78%) showed the same two allele-specific isoforms that were previously observed in LCLs in the same direction of effect, indicating that the AS event is also correlated with SNP genotype in this cell type. This indicates that for the majority of these cases, expression of allele-specific transcript isoforms occurs in a tissue-independent manner for the two tissue types that were tested, providing further support for a common transcriptional regulatory mechanism for these genes in HObs and LCLs.

**Table 1:** Replication of validated LCL probesets in Hobs.

Gene	Probeset ID	SNP (LCL)	P-value (LCL)	SNP (HOb)	P-value (HOb)	Replicated in HObs?
Cep192	3779862	rs482360	3.71E-19	rs570505	4.22E-03	No
ZNF83	3869658	rs1012531	2.72E-10	rs1012531	5.39E-02	Yes
C17orf57	3724617	rs3760372	5.54E-12	rs3760372	4.29E-01	No
CAST	2821249	rs7724759	7.17E-16	rs13362120	4.73E-13	Yes
CD46	2377476	rs4844390	1.06E-14	rs2761437	6.02E-02	Yes
FLJ10241	3863093	rs1043413	9.38E-11	rs3810174	1.15E-03	Yes
LRAP	2821389	rs2255546	8.37E-22	rs2161657	8.39E-01	Yes
POMZP3	3057764	rs2005354	3.77E-22	rs17718122	1.70E-10	No
ULK4	2670619	rs1717020	5.99E-11	rs9852303	1.45E-05	Yes
PARP2	3527423	rs2297616	2.81E-37	rs3093942	4.94E-07	Yes
ATPIF1	2327383	rs2481974	4.26E-11	rs8559	1.70E-01	No
MRPL43	3303658	rs12241232	1.24E-11	rs3740487	2.44E-05	Yes
DKFZp451M2119	2588913	rs10930785	1.93E-28	rs10930785	1.56E-09	Yes
RNH1	3358076	rs11821392	4.34E-15	rs12420868	4.88E-01	Yes
SNX11	3725089	rs7224014	4.20E-09	rs2240122	1.47E-01	No
USMG5	3304753	rs7911488	2.66E-24	rs1163073	6.45E-11	Yes
SEP15	2421300	rs1407131	7.57E-13	rs17452535	1.01E-02	Yes
SLC35B3	2941033	rs3799255	2.12E-10	rs3799255	1.30E-02	Yes
DERP6	3708382	rs2521985	2.55E-13	rs222851	6.18E-04	Yes
ARTS-1	2868133	rs7705827	6.09E-19	rs1862609	3.98E-06	Yes
TAP2	2950168	rs3763355	1.98E-13	rs241448	4.48E-01	No
IRF5	3023264	rs6969930	8.27E-22	rs10239340	8.94E-01	Yes
PPIL2	3938301	rs5999098	1.46E-12	rs5754727	1.54E-02	Yes
PTER	3236819	rs1055340	5.25E-18	rs7909832	6.24E-13	No
WARS2	2430765	rs1325933	3.53E-08	rs1325933	6.38E-01	Yes

List of candidate probesets previously validated in Kwan et al (2008) by qualitative or quantitative RT–PCR. The gene name and the significant probeset are indicated along with the SNP in LCLs and P-value from the linear regression analysis. Since the HOb panel was only genotyped using the Hap550K chip, we selected the SNP in HOBs that was in highest LD with the original SNP associated in LCLs. The last column indicates whether the allele-specific isoform changes were originally validated in LCLs was replicated in the osteoblast samples.

### Tissue-specific genetic variation of transcript isoforms

Next, we examined the set of genes expressed in both cell types (n = 7733) for examples of tissue-specific genetic associations, i.e. allele-specific expression of isoform variants in HObs but not in LCLs, to identify any unique transcript isoforms with potentially important biological functions in osteoblasts. One criterion was that the gene itself was not significantly associated (P>0.05) with the same SNP as the probeset, in order to limit the number of probesets within the gene showing allele-specific expression differences and also to reduce the complexity of interpreting the predicted isoform changes. The top 100 ranked unique probesets (P<1.338e-5) in HObs corresponded to 84 unique meta-probesets. To identify allelicly expressed probesets specific to HObs but not to LCLs, the association analysis for the same probeset and SNP in LCLs was performed and any probeset showing a nominal P-value<0.05 in LCLs was discarded. This resulted in 48 probesets from 47 unique meta-probesets showing significant HOb-specific associations. Conversely, we also selected the top 100 unique probesets (P<2.776e-7) in LCLs corresponding to 82 meta-probesets. These top ranked probesets in LCLs were then filtered to exclude for significance in HObs, resulting in 40 probesets from 34 meta-probesets that showed LCL-specific genetic association. These results indicate that nearly half of the top 100 ranking hits in HObs are not significant (nominal P-value<0.05) in LCLs, and this frequency is similar when comparing the top LCL associations to the HObs, suggesting that we can identify tissue-specific in addition to tissue-independent isoform variations from the microarray data.

We selected 20 examples of tissue-specific isoforms showing large effect sizes in one tissue but not the other, for validation by standard RT-PCR and electrophoresis, however we only observed the similar expression patterns in both tissues, indicating a lack of tissue-specificity. Since this is a qualitative-based detection method, we used the more sensitive quantitative real time RT-PCR to detect possible cases of smaller expression differences between isoforms for another set of candidates. We looked at an additional seven HOb-specific candidate isoforms events, and in four cases (*TIPARP*, *ESPNL*, *P4HA2*, and *EPN3*) we observed allele-specific expression differences in osteoblasts but not in lymphoblasts, using cutoffs of significance and non-significance in these two tissues respectively, as described in the Materials and Methods. *P4HA2* is a prolyl 4-hydroxylase which is a key enzyme in collagen synthesis, and *ESPNL* and *EPN3* are part of the espin gene family that are involved in the production of the extracellular matrix. The identification of transcript isoform variants in one tissue but not the other indicates that there are some tissue-specific factors at play, either in promoting transcription of the SNP-associated isoform or suppressing the genetic effect of the polymorphism. However, our low validation rate of the microarray results suggests that *cis* variants controlling expression of tissue-specific isoforms may not contribute to a large proportion of inter-tissue variability.

The set of genes uniquely expressed in HObs showing genetic associations were examined for any enrichment in biologically relevant functions. While this biases the analysis towards genes having a potentially important function in osteoblasts, the aim was to assess whether certain genes or entire pathways are preferentially under the influence of genetic control. The 1000 highest ranked unique probesets showing SNP association were examined using IPA and the top canonical pathways identified (Table S6) included the Wnt/β-catenin signaling system (P = 2.97e-3) and IGF-1 signalling pathways (P = 2.63e-2) important in the bone remodeling process [22],[23],[24]. Conversely, examining the top 1000 ranked probesets in lymphoblasts revealed the B cell receptor signaling (P = 1.32e-4), iCOS-iCOSL Signaling in T Helper Cells (P = 1.45e-3), FcγRIIB Signaling in B Lymphocytes (P = 1.88e-3), *BRCA1* in DNA damage response (P = 4.02e-3), and Fc Epsilon RI signaling (p = 1.09e-2) among the top LCL networks associated with potential isoform variation.

### Comparison of probeset associations with known bone-related GWAS loci

A recent GWAS [16],[17] with 300K SNPs identified sequence variants in nine genomic regions significantly associated with bone mineral density (BMD), which influences the risk of osteoporosis. We examined the top 100 SNPs from quantitative trait analyses of hip BMD [17], of which 91 were tested in our analysis, and looked at the relative levels of replication for these polymorphisms in our probeset association results for potential transcript isoform variations linked to the same SNPs or to a SNP in close LD (defined as D' = 1, MAF>0.10 and located ±50 kb flanking the GWAS SNP). In our HOb probeset associations, we observed 15 out of 91 (16%) BMD GWAS SNPs showing association at a Bonferroni cutoff of 5.49e-04 (0.05/91) (Table S9). This indicates that of the top polymorphisms identified from GWAS, a small proportion of them may be involved in the regulation of alternatively spliced transcripts that play a role in modulating the BMD phenotype. These 100 SNPs are represented by 63 loci, and of these 63 loci implicated in BMD variation, 13 loci (21%) may be attributable to *cis*-regulatory variation.

### Validation of a novel bone-related GWAS locus: *FAM118A*

**Figure 1:**
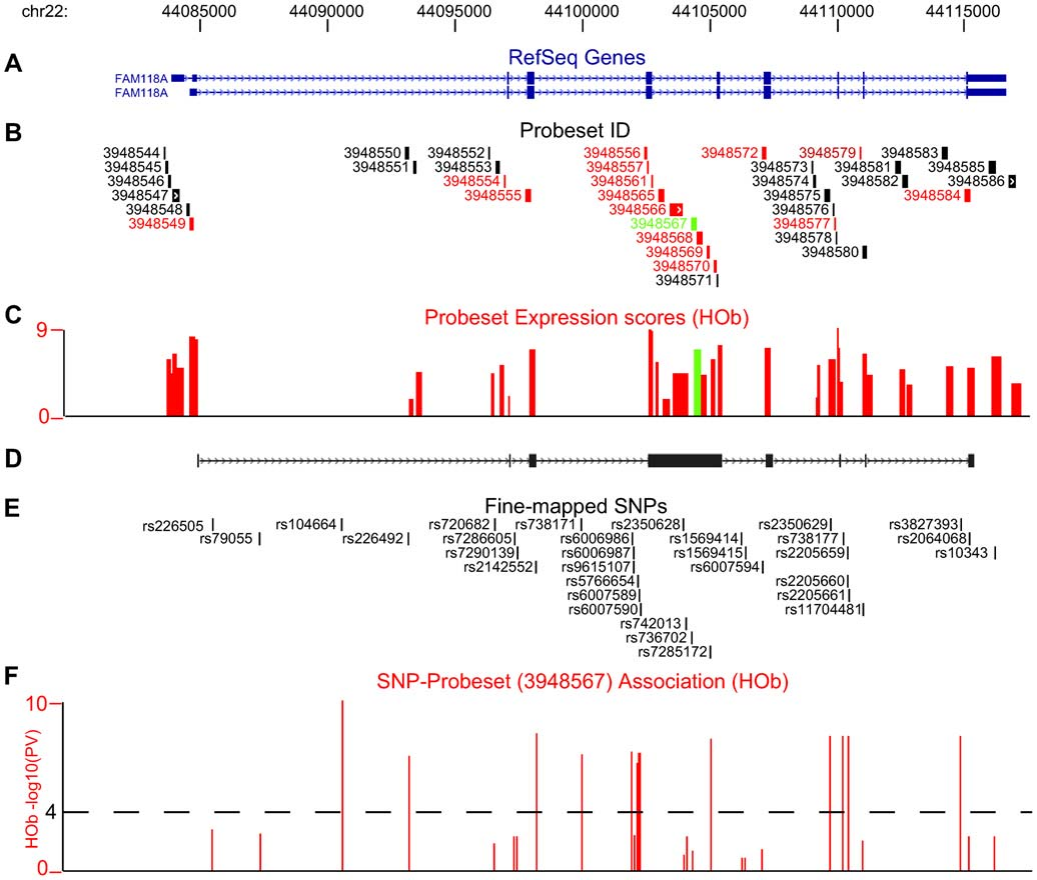
Validation of the genetic effect of *FAM118A* probeset expression. (A) RefSeq transcript variants, NM_001104595 and NM_017911, for the *FAM118A* gene and (B) all corresponding probesets included on the Affymetrix Human 1.0 ST Exon array. RMA normalized expression scores for all probesets, as shown by vertical bars (C), indicate expression of additional probesets that differ from the RefSeq variants. Validation studies by RT–PCR and 5′ RACE confirmed expression of probesets marked in red (A) and represent a novel *FAM118A* transcript variant (D). Fine-mapped SNPs (E) show highly significant associations with expression of probeset 3948567 (marked as green throughout the figure) and P-values represented as –log_10_(P-value) are shown as vertical bars (F). Dashed line indicates cutoff P = 10e-4.

**Figure 2:**
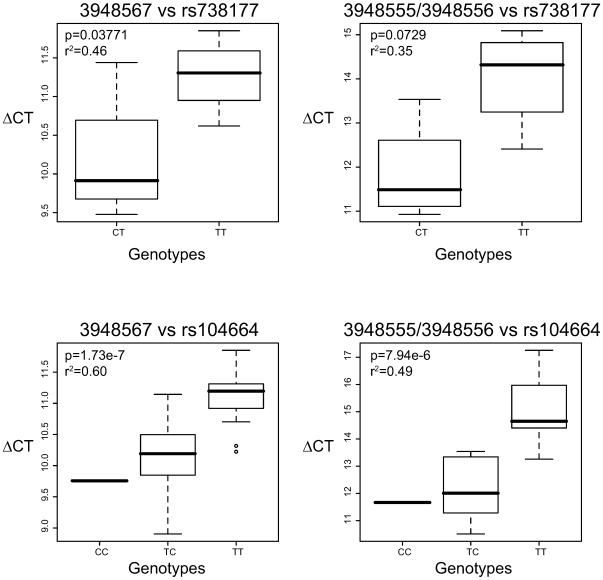
Validation of microarray association using real-time RT–PCR. The effect of SNPs on *FAM118A* probeset expression was validated by quantitative real-time PCR in HObs (n = 31, lower panel) and LCLs (n = 8, upper panel). The rs104664 and rs738177 represent the top significant SNPs associated with probeset expression in the microarray analysis in HObs and LCLs, respectively. Primers were designed to amplify probesets 3948567 (left panel) and 3948555/3948556 (right panel), respectively. Relative expressions were calculated using the comparative CT method using 18S as a housekeeping gene and associations were assessed using a linear regression model. The P-values and R^2^ of the linear regression statistic are shown within each boxplot.

Based on the comparison of probeset associations with known bone-related GWAS loci, we examined the top hit (rs136564, P = 4.88E-07, Table S9) in greater detail to determine the nature of a possible isoform variant. The probeset (3948557, chr22:44,102,552-44,102,577) maps to exon 5 in the *FAM118A* gene (Figure 1A) encoding a hypothetical protein (LOC55007) with so far unknown function. We fine-mapped (chr22:44,083,527–44,118,573) and re-sequenced (chr22:44,097,900–44,104,182) the candidate region and expanded the association analysis of transcript isoform variations by including non-core probesets (chr22:44,083,527–44,118,573) (Figure 1B). The non-core probesets are defined as the less confident probesets on the Exon Array corresponding mainly to computationally predicted exons rather than experimentally verified RefSeq exons. Using the information from all the *FAM118A* probesets (Figure 1B), we detected expression of additional probesets between exons 5 and 6 that differ from the RefSeq variants (NM_001104595 and NM_017911) (Figure 1C) suggesting a potentially new transcript isoform variant, previously unsupported by ESTs. We confirmed the novel transcript variant by sequencing RT-PCR and 5′ RACE amplified fragments (Figure 1D) from eight individuals (four samples from each homozygous genotype group of the top SNPs). The identified transcript variant differs from the RefSeq variants at the 5′UTR, exon 5 and at the 3′UTR, respectively (Figure 1D). We observed highly significant associations of fine-mapped SNPs (Figure 1E) (see Table S10 for full list of SNP-probeset associations) in the *FAM118A* gene with expression of probesets within the 46;1F). Notably, we did not observe associations of probesets uniquely expressed in the RefSeq variants (3948549, 3948585, data not shown). We then performed a similar association analysis using our LCL CEU samples but extended the analysis by including all HapMap SNPs in the region of interest (i.e. ±250 kb flanking the *FAM118A* transcript) and found a similar, highly significant association of expressed, transcript-specific probesets and SNPs within the *FAM118A* gene (Figure S3). However, the strong LD among the common SNPs associated with the probeset expression did not allow delineation of the causal variant(s). We confirmed the significance and direction of the association analysis of the *FAM118A* SNPs for both HObs and LCLs by quantitative real-time RT-PCR using two different primer pairs (Figure 2). As shown in Figure S4, the tissue independent association of probeset expression seen in HOb samples was further confirmed in a second LCL population, the Yoruba (YRI) population [5],[25], as well as in cortical brain tissue samples and peripheral peripheral blood mononuclear cells (PBMCs) from the SNPExpress Database [11].

To validate the original finding in the GWAS linking genetic variants in the *FAM118A* locus with differences in bone phenotype, we performed a candidate gene association study of BMD and our top candidate SNP (rs104664, see Figure 1) in a cohort of randomly selected elderly Swedish men from the MrOs Sweden study. In total, 2880 individuals were analyzed with genotype frequencies of: CC = 0.02, CT = 0.26 and TT = 0.72 (MAF C = 0.15, H-W P = 0.31). The candidate SNP was associated with lumbar spine BMD (P = 0.0065, n = 2856) but not with femoral neck BMD (P = 0.61, n = 2778). Lumbar spine BMD decreased 0.067 g/cm^2^ in the minor C allele homozygous group compared to the TT group. We pursued replication of the association of the rs104664 SNP with BMD in 2100 elderly men from the population-based Rotterdam study with genotype frequencies of: CC = 0.02, CT = 0.20, and TT = 0.78 (MAF C = 0.12, H-W P = 0.02). We were however not able to replicate the previous findings in the Rotterdam study either with lumbar spine (P = 0.35) or femoral neck (P = 0.28) BMD.

## Discussion

The study of transcriptomic effects of genetic variation is shifting from the use of well-characterized lymphoblastoid cell lines (LCLs) to primary cell lines that are more biologically relevant to a particular disease or phenotype of interest. Human osteoblasts (HOb) are the primary cell type responsible for the bone remodeling process and alterations in this pathway can lead to disorders such as osteoporosis, therefore HObs represent a very good model system for the study of bone biology and bone-related diseases in general. Using a strategy that we previously employed for the study of genetic variation in LCLs, we assessed SNP association in the HOb transcriptome to assess the nature of common and unique *cis*-variants controlling transcript isoform expression in the two cell types.

Gene ontology analyses of the top associations from each tissue also revealed certain networks that were enriched in these isoform variants. Genes that are expressed in multiple cell types are predicted to share many common biological functions and pathways responsible for general cell growth and survival. In fact, genes whose expression levels are very similar in both HObs and LCLs are enriched in pathways that are important in cellular growth and maintenance such as inositol phosphate metabolism, N-glycan synthesis, purine metabolism, and pyrimidine metabolism. Functions that are required for general cell survival across multiple tissue types are expected to be constitutively on and therefore should require less regulatory mechanisms and modules controlling its expression as compared to tissue-specific genes whose expression needs to be temporally and spatially controlled by a set of more complex regulatory sequences and events. The top osteoblast specific isoform variants preferentially mapped to biological networks involved in cell and tissue development, including tight junction signaling and keratan sulfate biosynthesis pathways suggesting more complex regulation of these functions.

A large set of genes is expressed in both HObs and LCLs, producing a vast array of transcript isoforms that are affected in both cell types by a common regulatory SNP and tissue-independent fashion. However, certain isoform variations are tissue-specific despite the presence of the same genetic background or haplotype in other cell types, suggesting that other mechanisms, such as tissue-specific transcription factors, splicing factors, or small non-coding RNAs that are present in only one of the cell types additionally regulates transcription. For example, brain tissue expresses a neuronal-specific splicing factor, Nova, which increases the levels of alternative splicing in neuronal versus immune tissues, as shown from recent studies using Nova knockout mice [26]. Despite the presence of identical regulatory sequences in neuronal and immune tissues and in both wild-type and knockout mice, only these sequences in brain are recognized by Nova due to its restricted expression pattern, resulting in the generation of multiple brain-specific isoforms.

Despite the limited number of validated examples, osteoblast-specific transcript isoforms are present and indicates that expression of certain alternatively spliced variants in bone tissue are controlled by different regulatory SNPs and vary between individuals and/or populations, with potentially important biological functions. The future use of more sensitive quantitative methods may identify smaller, subtler differences in isoform expression that can alter an important biological pathway, leading to a cascade of downstream functional consequences. A plausible explanation is that the combined effects of many such genetic variations are probably necessary for deviations in the normal function of complex diseases such as osteoporosis.

One factor compounding an accurate statistical comparison of genetic variation between the different cell types were the numbers of samples used: 58 HObs (n = 1) versus 57 LCLs (n = 3). The triplicate LCLs decreases variability between samples compared to the single replicate HOB samples; therefore linear regression with the noisier HOb data set will result in lower significance in P-values for the regression statistic. The small sample sizes used here for each tissue type is also limiting in the power to detect very large size effects, even for very significant genetic associations, and is a caveat to the identification and validation of many but the top associations, but at the benefit of reduced cost for the experiment. Another important factor contributing to the relatively little overlap is due to expression level differences between tissues. Generally, genes with a strong genetic expression effect and with high expression in LCLs will be highly ranked by P-value significance from the linear regression analyses. However, if the expression signal is much lower and therefore signal strength is noisier in HObs, despite an equally strong genetic effect, the linear regression statistic produces a lower P-value and hence, a lower ranking on our list. A comparison of the top 100 significance-ranked probesets from similarly expressed genes versus genes with at least 3-fold expression differences between the two tissues showed a level of replication that was nearly two-fold higher in the set of genes with comparable expression (data not shown). This explains the low level of replication between the tissues from the microarray data analyses and suggests that microarray results may in fact underestimate the extent of genetic variation common to both tissues. Since gene expression is also highly variable from tissue to tissue, the sensitivity of microarray studies for any given subset of genes will also vary for any given tissue, compounding the analysis of common variation. These differences in dynamic range between the methods used may explain some of the contrasting results for the levels of replication between LCLs and HObs that we observed using microarrays or RT-PCR validation. The discrepancy between associations based on the microarray results and the RT-PCR validation studies can also be explained by the false positive rates associated with the analysis of microarray data. If one assumes a liberal false positive rate of 50% from one tissue type, combining the analysis with potential hits from a 2^nd^ tissue type with an equal false positive rate to look for tissue-specific hits, one can expect a maximum of 25% true positives.

Our results were compared with a similar study examining tissue-specific genetic control of splicing in brain tissues and PBMCs. Using very stringent cutoffs, 23 and 80 high confidence associations at the meta-probeset and probeset level, respectively were identified. However, fewer than half the associations were common to both tissues, reflecting strong evidence for distinctive tissue-specific genetic control of transcript isoform expression. This corroborates the suggestion of tissue-specificity of genetic control from our own SNP-association results in HObs and LCLs. One contrasting difference was that our rate of validation did not strongly support the microarray data as we would have expected. Three examples of tissue-independent and tissue-specific genetic control of splicing (*ULK4*, *SLC12A7*, *KLHL24*) were shown, however these were not the most significant candidates from the study, nor was any information provided on validation rates. Without rigorous validation of more candidate splicing events from both studies, we still cannot assess the prevalence of genetic control of tissue-specific transcript isoforms. Moreover, the assessment of tissue-specific transcript isoforms in samples derived from different individuals reported here and by others [11] is a limitation since small differences in allele frequencies can bias the comparison. It is only through examination of multiple tissues derived from the same individual that we can obtain the full spectrum of tissue-specific genetic variation of transcript isoforms.

Comparison of our exon-level eQTL data with known SNPs from a GWAS of bone mineral density indicates that ∼20% of the top associated SNPs from the quantitative trait study may be linked to transcript isoform variation. Follow up of one of the top hits (*FAM118A*) from our association data that overlaps with the GWAS hits revealed a novel isoform variant associated with three SNPs obtained from fine-mapping the gene region. Further analysis in different tissues and populations confirmed the strong association of SNPs with expression of the novel transcript variant.

The osteoblast donors were not ascertained for osteoporosis or BMD to allow testing directly the transcriptomic association at the population level. However, the genetic contribution to trait variance of cell-based traits are several-fold higher as compared to complex traits assessed in clinical cohorts, so even if phenotypes were available for the osteoblast donors, our power would have been low in detecting small differences. Instead, we tested the top hit in *FAM118A* in an independent Swedish cohort of >3000 men where we found the SNP to be associated with the BMD phenotype. Individuals homozygous for the minor C allele, earlier shown to be associated with increased expression of the novel isoform, had lower BMD indicating a negative effect of the expression of the gene on bone phenotypes. The failure to replicate the association in the second cohort is probably due to the lack of power needed to detect such small genetic effects (B = 0.63), as shown in a *post-hoc* power analysis using α = 0.05 and an effect size explaining 0.005 of the additive QTL variance in BMD. Other factors like population specificity due to differences in LD, differences in phenotype assessment, selection of individuals and/or genotyping errors may also play a role. We cannot rule out that some of the discrepancy in association between the two cohorts may be due to a false positive result, however this will require further extensive validation in both cohorts.

There is an increasing trend amongst the scientific community towards the use of primary tissues from normal and disease-affected patients for the association and fine mapping of genetic variants with quantifiable phenotypic traits. These studies are being carried out in increasingly large cohort samples that are more costly for the collection, processing, and microarray analyses of these samples. Despite the limitations of using the HapMap LCLs as described earlier, they do possess the advantage of availability and wealth of public data derived from these samples. Our data comparing LCLs and HObs, in particular the RT-PCR replication of many of our LCL isoform variants in osteoblasts, suggests that although we can identify HOb-specific transcript variants, a large proportion of variation for genes expressed in both cell types is common between them. Studying transcriptome variation across multiple tissue types, and in particular the subsets of genes that are commonly expressed in all tissues, will allow us to determine the proportion of genetic variation that is common between different cell types. However, examining the sets of genes uniquely expressed in a single cell type will allow for the identification of cell-specific isoform variants that may be functionally important. Only through an examination of as many tissues as possible can we obtain the full spectrum of genetic variation in the human transcriptome. Using these large expression and association datasets in conjunction with the expanding list of polymorphisms identified from GWAS may aid in the fine mapping and delineation of the molecular basis behind various diseases and quantitative phenotypes.

## Materials and Methods

### Bone cell culture for whole genome expression profiling

Human trabecular bone from the proximal femoral shaft was collected from 60 donors undergoing total hip replacement. The bone samples from each donor were thoroughly washed, minced and cultured in medium containing α-MEM (Sigma-Aldrich, Oakville, Canada) supplemented with 2 mmol/l L-Glutamine, 100 U/mL penicillin, 100 mg/mL streptomycin (Invitrogen, Burlington, Canada), and 10% fetal bovine serum (Sigma-Aldrich, Oakville, Canada) at 37°C with 5% CO_2_ until confluence was reached. At 70–80% confluence, the cells were harvested and stored in −70°C until RNA and DNA extraction.

### RNA and DNA extraction

RNA was isolated using the commercially available TRIZOL reagent (Invitrogen, Burlington, Canada) protocol and treated with DNaseI (Ambion Inc., Austin, TX, USA) for 40 min at 37°C and further extracted with phenol/chloroform (Invitrogen, Burlington, Canada). The RNA was further purified using the RNeasyMinElute Cleanup kit (Qiagen, Mississauga, Canada). High RNA quality was confirmed for all samples using the Agilent 2100 BioAnalyzer (Agilent technologies, Palo Alto, CA, USA) and the concentrations were determined using the Nanodrop ND-1000 (NanoDrop Technologies, Wilmington, DE, USA). DNA was extracted from the cell lysates using the GenElute DNA Miniprep Kit (Sigma-Aldrich, Oakville, Canada) according to the protocol provided by the manufacturer.

### Microarray analysis

Expression profiling was performed for each sample with the Affymetrix Human 1.0 ST Exon array (Affymetrix, Santa Clara, CA). One microgram of RNA was reverse transcribed into cDNA, and in vitro transcription was performed to generate biotin-labeled cRNA for subsequent hybridization. Hybridized target cRNA was then stained with streptavidin-phycoerythrin, and the arrays were scanned with a GeneArray Scanner at an excitation wavelength of 488 nm. The microarray data have been deposited in the Gene Expression Omnibus (GEO; www.ncbi.nlm.nih.gov/geo, accession no. GSE15252).

### Robust multichip average (RMA) adjustments

All probeset and meta-probeset expression values from the human primary bone samples were generated using the robust multichip average (RMA) algorithm from the Affymetrix Power Tools software package. To prevent the introduction of batch effect bias between the osteoblast and lymphoblast datasets, the raw microarray data from both tissue sample panels were quantile normalized together. All probes that have an overlap to a SNP from dbSNP release 126 were masked out from the probeset summarization procedure to reduce the influence of polymorphisms on probe hybridization to a SNP-containing target [27]. Meta-probeset expression values were generated from the probeset expression summaries.

### Genotyping of samples

Genotyping was performed using the Illumina HapMap 550 k Duo chip (Illumina Inc, San Diego, CA) according to protocols provided by the manufacturer. The total number of SNPs included on the chip is 561,303. Two individuals with low genotyping rate were excluded (array call rate<98%), as were 90401 SNPs with low frequency (minor allele frequency<5%), high rates of missing data (SNP call rate<90%), and showing significant deviation from Hardy-Weinberg equilibrium (P<0.05), leaving 58 samples for the study.

### Transcriptomic Association Analysis

Probeset (exon) and meta-probeset (gene) expression levels were examined for association with flanking SNPs. For each of the 274,097 core probesets and 17,749 meta-probesets, we tested for association of the expression levels to SNPs within a 250 Kb region flanking either side of the gene containing the probeset or meta-probeset, using a linear regression model implemented in the PLINK software package [28]. Gender, age, batch effects, and eigen values for the 1^st^ axis from the Eigenstrat analysis were used as covariates in the regression model. Raw P-values were obtained from the regression using the standard asymptotic t-statistic.

### Networks Analysis

To visualize whole genome expression data in the context of biological networks, we created meta-probeset lists based on expression cutoffs and meta-probeset or probeset lists from top-ranked SNP associations in one or both cell types, which were then analyzed with the Ingenuity Pathways Analysis (IPA) system (Ingenuity Systems, www.ingenuity.com). Datasets containing the meta-probeset or probeset IDs and their corresponding expression values or minimal P-values were uploaded in the application and were mapped to its corresponding gene object in the Ingenuity Pathways Knowledge system. These genes were overlaid onto a global molecular network developed from the Ingenuity Pathways Knowledge Base. IPA datasets for all species, all tissues & cell lines, and all data sources were used in the analysis with default settings. Networks of these genes were then algorithmically generated on the basis of their connectivity.

The functional analysis identified the biological functions that were most significant to the data set. Only genes that met the cutoff and were associated with biological functions in Ingenuity Pathways Knowledge were considered for analysis. Fisher's exact test was used to calculate a P-value determining the probability that each biological function assigned to the data set is due to chance only.

Canonical pathways analysis identified the networks that were the most significant to the data set. Genes that met the cutoff in the functional analyses and were associated with a canonical pathway in the Ingenuity Pathways Knowledge Base were considered for the analysis. The significance of the association between the data set and the canonical pathway was measured in two ways: 1) a ratio of the number of genes from the data set that map to the pathway divided by the total number of genes that map to the canonical pathway was displayed and 2) Fisher's exact test was used to calculate a P-value determining the probability that the association between the genes in the data set and the canonical pathway is explained by chance alone.

### Validation of transcript isoform changes by RT-PCR

First strand cDNA was synthesized using random hexamers (Invitrogen, Burlington, Canada) and Superscript II reverse transcriptase (Invitrogen, Burlington, Canada). Candidate probesets showing an association were validated in two ways, depending on their location within the gene. For all probesets located within coding exons and possessing flanking exons in all known RefSeq isoforms, we designed locus specific primers within the adjacent flanking exons. Approximately 20 ng of total cDNA was then amplified by PCR using Hot Start Taq Polymerase (Qiagen, Mississauga, Canada) with an activation step at 95°C (15 min) followed by 35 cycles at 95°C (30 s), 58°C (30 s) and 72°C (40 s) and a final extension step at 72°C (5 min). Amplicons were visualized by electrophoresis on a 2.5% agarose gel.

For probesets located within the 5′ or 3′ untranslated regions or within exons that did not have both flanking exons (i.e. first or last exon of a multi-exon gene), we designed a set of primers to amplify the differentially expressed candidate probeset itself. For comparison, additional primer pairs were designed to amplify products corresponding to the adjacent probesets and which were not significantly associated with the same SNP. Total expression measurements were carried out using real-time PCR with Power SYBR Green PCR Master Mix (Applied Biosystems, Foster City, CA, USA) following the manufacturer's instruction on an ABI 7900HT (Applied Biosystems, Foster City, CA, USA) instrument. The reaction was set up in 10 µl final volume applying the following conditions: 8 ng of total cDNA and 0.32 µM of gene-specific primers; cycling: 95°C (15 min) and 95°C (20 s), 58°C (30 s), 72°C (45 s) for 40 cycles. Relative quantification of each amplicon was evaluated on RNA from at least 3–4 samples corresponding to each of the three possible genotypes for the associated SNP. For each amplicon including the human 18S used as endogenous control, a standard curve was established using a dilution series of a mix of cDNA samples with known total cDNA concentration in order to determine if the amplification reactions had the same PCR efficiency. Expression levels of target and 18S were evaluated using the comparative Ct (cycle threshold) method where three technical replicates were averaged and normalized based on 18S real-time data from the same samples. The quantitative data was used in regression analyses with the same SNP identified in the original association to confirm the significance, using a P-value threshold of 0.05/N where N is the number of candidate genes tested using this method. The regression trend had to be in the same direction as in the original association. Quantitative RT-PCR of the control probesets showing no association with the SNP should also be non-significant at this threshold.

### 5′ Rapid Amplification of cDNA Ends (RACE)

The 5′ Rapid Amplification of cDNA Ends (RACE) System (Invitrogen, Burlington, Canada) was used to identify the unknown sequences at the 5′ end of the novel *FAM118A* transcript according to manufacturer's guidelines. First strand cDNA was synthesized from 1 µg of total RNA using a gene-specific primer and SuperScriptII reverse transcriptase (Invitrogen, Burlington, Canada). The original mRNA template was removed by RNase H treatment and the first strand cDNA product was purified on columns provided with the system to remove unincorporated dNTPs and primers. A homopolymeric tail was added to the 3' end of the cDNA using TdT and dCTP, and the fragment was amplified by PCR using a nested, gene-specific and a deoxyinosine-containing anchor primer provided with the system using Hot Start Taq Polymerase (Qiagen, Mississauga, Canada) with an activation step at 95°C (15 min) followed by 35 cycles at 95°C (30 s), 58°C (30 s) and 72°C (3 min) and a final extension step at 72°C (10 min). The PCR product was diluted 50×in TE buffer and further amplified using a nested gene-specific primer and an Abridged Universal Amplification Primer provided with the system using Hot Start Taq Polymerase with an activation step at 95°C (15 min) followed by 30 cycles at 95°C (30 s), 58°C (30 s) and 72°C (3 min) and a final extension step at 72°C (10 min). Amplicons were visualized by electrophoresis on a 1% agarose gel and sequenced using ABI Big Dye chemistry and capillary electrophoresis on an ABI 3730 sequencer (Applied Biosystems, Foster City, CA).

### Fine-mapping of *FAM118A*

The top hit from the comparison of probeset associations with known bone-related GWAS loci was further fine-mapped where selected HapMap SNPs in the *FAM118A* (Table S7) gene (chr22:44,083,527–44,118,573) were genotyped using the Sequenom MassARRAY iPLEX Gold technology (Sequenom Inc., Newton, MA). Briefly, around 40 SNPs were multiplexed per individual reaction and a single base primer extension step was performed. The primer extension products were then analyzed using MALDI TOF MS.

### Re-sequencing and genotyping of non-HapMap SNPs

To identify all genetic polymorphisms potentially responsible for the differences in gene expression, approximately 6 kb of the candidate region (chr22:44,097,900–44,104,182) in the *FAM118A* gene was re-sequenced using standard Sanger sequencing using ABI Big Dye chemistry and capillary electrophoresis on an ABI 3730 sequencer (Applied Biosystems, Foster City, CA). In total, eight individuals were selected based on the candidate SNPs (four samples from each homozygous genotype group of the top SNPs) and overlapped regions were amplified and sequenced using primers listed in Table S8. Non-HapMap SNPs and/or novel SNPs that were homozygous in all eight samples were genotyped in the complete panel of HObs using Sanger sequencing.

### Primer design

Primers were designed using the Primer3 v. 0.4.0 software (http://frodo.wi.mit.edu/) and all primer sequences can be found in Table S8.

### Clinical cohorts

The effect of the identified SNPs on clinical bone phenotypes was tested in two population-based studies involving Caucasian male subjects of European origin: The MrOs Cohort, Sweden (MrOs), and The Rotterdam Study (RS), Netherlands.

The MrOs study is a multi-centre prospective fracture epidemiology investigation involving elderly men from different sites around the world including the US, Hong Kong and Sweden. The Swedish part consists of 3014 men aged between 69–81 years that were randomly selected from the population registry [29]. Bone mineral density (BMD) of the lumbar spine (L1-L4), and femoral neck was measured using dual energy X-ray absorptiometry (DXA) using either a Hologic QDR 4500/A-Delphi (QDR 4500 W, Hologic, Inc.) or a Lunar Prodigy DXA (GE Lunar Corp.). To allow pooling of DXA measurements performed with different equipments, standardized BMD (sBMD) was calculated using previously reported algorithms [30],[31]. DNA samples from 2880 individuals were included in the study and the rs104664 SNP was genotyped using the Sequenom MassARRAY iPLEX Gold technology (Sequenom Inc., Newton, MA) that includes a single base primer extension and MALDI TOF Mass Spectrometry.

The RS is a prospective population-based cohort study of chronic disabling conditions in Dutch elderly individuals age 55 years and over [32],[33]. BMD measurement at the lumbar spine (L1-L4) and the femoral neck was performed by DXA following standard manufacturer protocols (GE-Lunar Corporation, Madison, WI or Hologic Incorporated, Bedford, MA). The rs104664 SNP was obtained from microarray genotyping performed in the whole original RS cohort using the Infinium II HumanHap550K Genotyping BeadChip version 3 (Illumina) as part of a large population-based project on genetics of complex traits and diseases. Genotyping procedures were followed according to Illumina manufacturer's protocols. All participants of the original RS cohort with proper quality DNA samples (n = 6449) were genotyped with the array. Intensity files were analyzed using the Bead Studio Genotyping Module software v.3.1.14. A no-call threshold of 0.15 was applied to a custom-generated cluster file derived from the Illumina-provided cluster file (based on the cluster definitions applied to the HapMap CEPH cohort). In the custom- cluster file, 2308 SNPs with Genecall scores<0.90 were visually checked by two observers and manually re-clustered or zeroed accordingly. Poorly performing samples with low call rate and 10th percentile Genecall scores were excluded prior to calling genotypes. Any samples with a call rate below 97.5% (n = 209), excess autosomal heterozygosity>0.336 ∼FDR<0.1% (n = 21), mismatch between called and phenotypic gender (n = 36), or if there were outliers identified by the IBS clustering analysis deviating>3 standard deviations away from the population mean (n = 102), or with IBS probabilities>97% (n = 129) were excluded from the analysis; in total, 5974 samples were analyzed. Of the 5974 individuals with a mean age of 69.4 (SD 9.1) years, 3547 (59.4%) were women.

All statistical analyses for the clinical associations in the MrOs Study were performed using SAS 9.1 software (SAS institute Inc.). A general linear model was used to study the effect of genotype on phenotypes (lumbar spine and femoral neck BMD) adjusted for age, weight, height and study center. The RS used age- and weight-adjusted standardized residuals of both lumbar spine and femoral neck BMD analyzed under an additive (per allele) genetic model using PLINK 1.05

## Supporting Information

Figure S1 Principal Component Analysis (PCA) of lymphoblast and osteoblast samples. (A) A two-dimensional plot of the meta-probeset data showing the separation of the LCLs (n = 171) and HObs (n = 58). The percentage of variance attributed to principal components one and two are shown on the X and Y-axes, respectively. (B) A two-dimensional plot of Eigenstrat analysis of the HObs meta-probeset data.
(0.67 MB PDF)

Figure S2 False Discovery Rate (FDR) analysis of associations. FDR discovery analysis of association P-values. In order of decreasing significance, consecutive windows of 100 associations are extracted in one tissue. The corresponding P-values in the 2^nd^ comparative tissue are extracted and FDR is performed on this 2^nd^ set of P-values. The number of hits falling below the FDR cutoff (0.05 level = red, 0.01 level = blue) is plotted for each of these windows. This was done for meta-probesets in (A) HObs and (B) LCLs, as well as probesets in (C) HObs and (D) LCLs.
(0.32 MB PDF)

Figure S3 Tissue-independent *cis*-associations of FAM118A transcript expression. (A) The top significant SNPs associated with FAM118A transcript expression in different populations and tissues. P-values of association for probeset 3948567 expression scores and SNP genotypes from (B) HOb, (C) HapMap CEU LCL, (D) HapMap YRI LCL (Zhang et al, AJHG 2008), (E) PBMCs (Heinzen et al., PLoS Biology 2008), and (F) Cortical brain tissue (Heinzen et al., PLoS Biology 2008) are shown as vertical bars and represented as -log_10_PV. (G) RefSeq transcripts in the FAM118A and flanking regions. (H) YRI and (I) CEU linkage disequilibrium blocks.
(1.30 MB PDF)

Figure S4 Association of the top significant SNP with all FAM118A probesets in different populations and tissues. (A) All probesets corresponding to the newly identified FAM118A transcript variant. P-values of association for linear regression of the FAM118A probesets in different samples and genotypes of (B) rs104664 in HObs, (C) rs738177 in HapMap CEU LCLs, (D) rs742014 in HapMap YRI LCLs, (E) rs104664 in PBMCs, and (F) Cortical brain tissue are shown as vertical bars and represented as -log_10_PV. (G) Two different RefSeq transcripts of FAM118A, NM_001104595 and NM_017911.
(0.28 MB PDF)

Table S1 HOb panel information.
(0.02 MB XLS)

Table S2 Mean expression scores and fold change for HOb and LCL expressed genes.
(1.21 MB XLS)

Table S3 HOb and LCL expressed genes (canonical pathways).
(0.02 MB XLS)

Table S4 Canonical pathways for tissue-specific expressed genes.
(0.02 MB XLS)

Table S5 Canonical pathways of tissue-specific upregulated genes (expressed in both HObs and LCLs).
(0.02 MB XLS)

Table S6 Canonical pathways of tissue-specific genetic associations at probeset level.
(0.02 MB XLS)

Table S7 Fine-mapped SNP of the FAM118A locus.
(0.02 MB XLS)

Table S8 Primer sequences.
(0.03 MB XLS)

Table S9 Probeset associations with known bone-related GWAS loci.
(0.05 MB XLS)

Table S10 Association of SNPs in the FAM118A gene with probeset expression.
(0.14 MB XLS)
