# Supplementary figures and images for: Tissue Effect on Genetic Control of Transcript Isoform Variation
Source: PLoS Genet. 2009 Aug 14;5(8):e1000608. doi: 10.1371/journal.pgen.1000608 (PMC2719916; doi:10.1371/journal.pgen.1000608)

**A****PCA component 2 vs 1, All**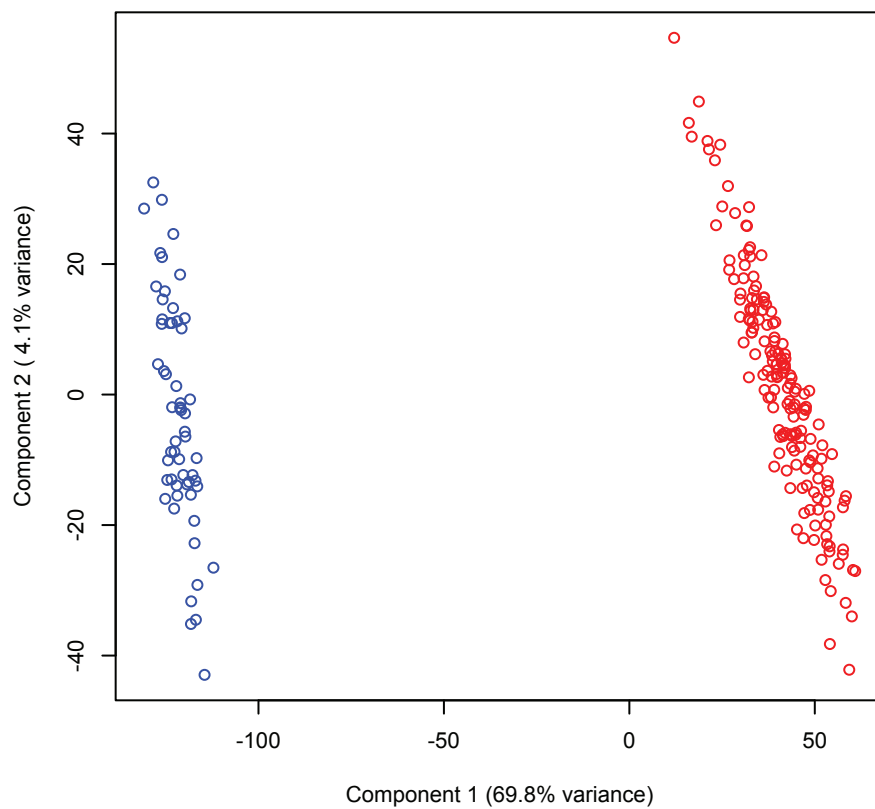**B**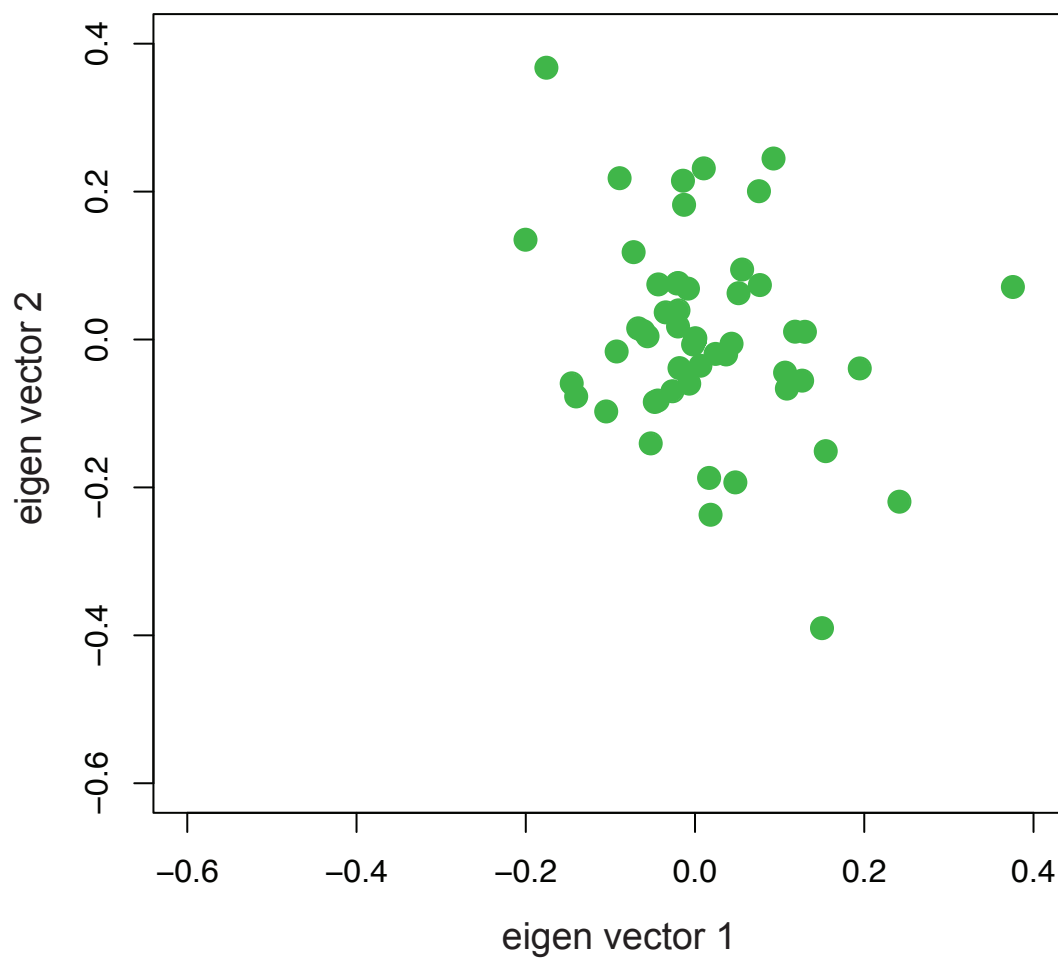

Supplement: Figure S1 — Principal Component Analysis (PCA) of lymphoblast and osteoblast samples. (A) A two-dimensional plot of the meta-probeset data showing the separation of the LCLs (n = 171) and HObs (n = 58). The percentage of variance attributed to principal components one and two are shown on the X and Y-axes, respectively. (B) A two-dimensional plot of Eigenstrat analysis of the HObs meta-probeset data. (0.67 MB PDF) [file pgen.1000608.s001.pdf]

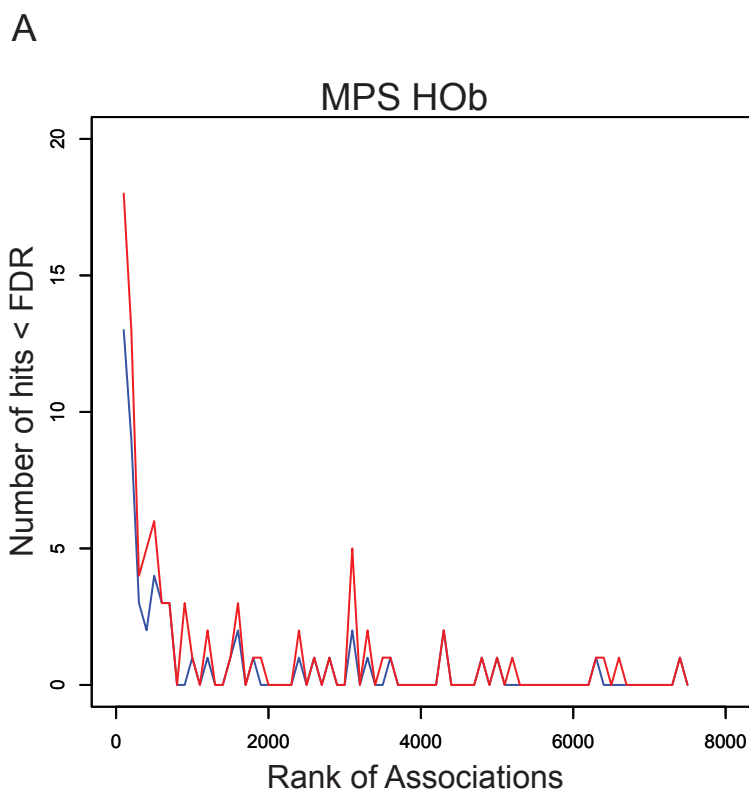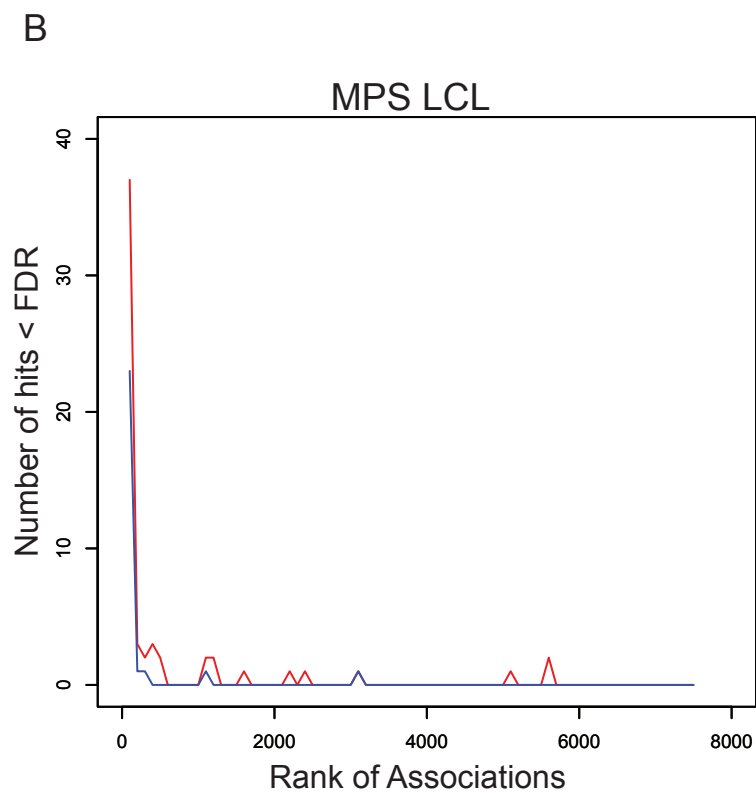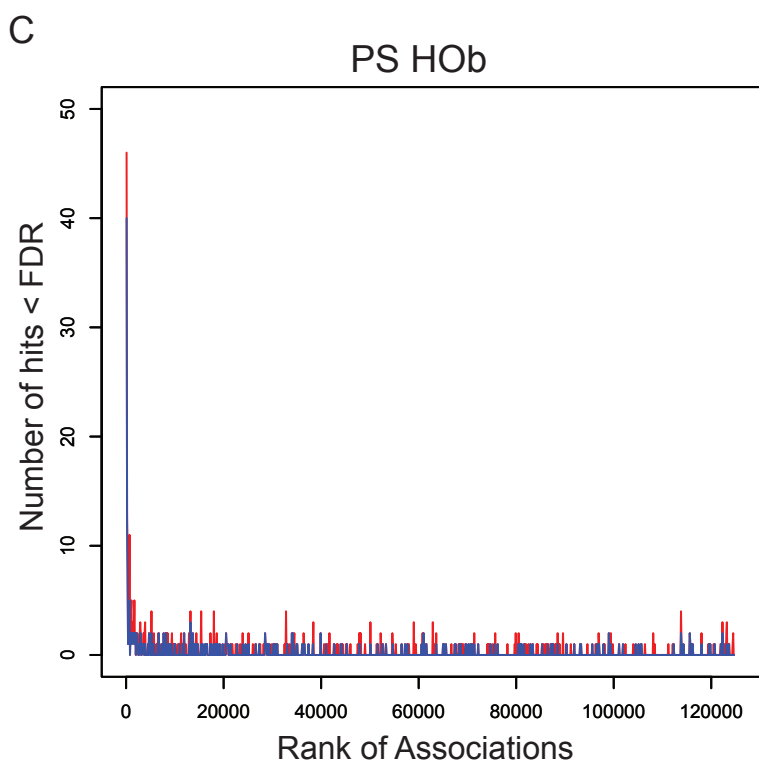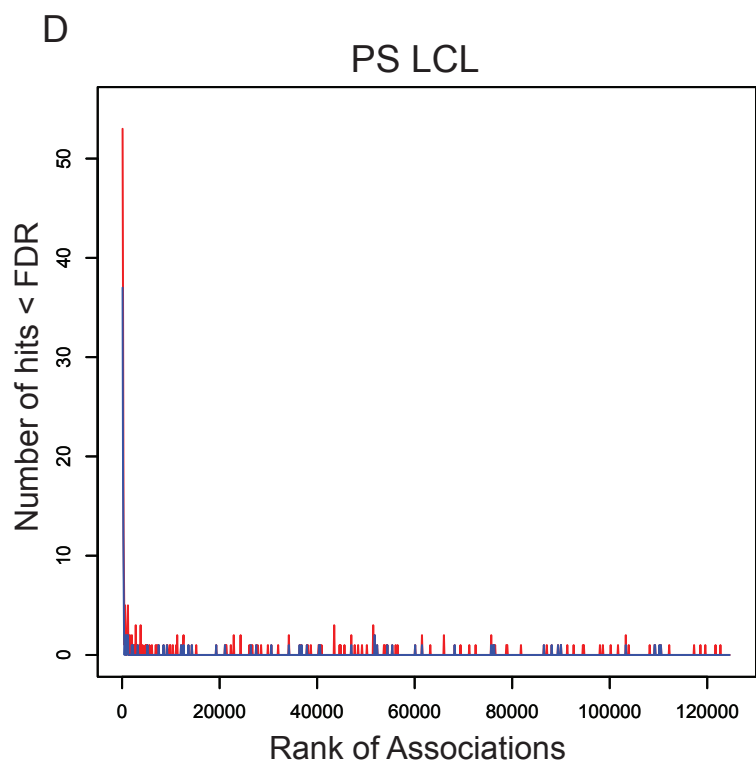

Supplement: Figure S2 — False Discovery Rate (FDR) analysis of associations. FDR discovery analysis of association P-values. In order of decreasing significance, consecutive windows of 100 associations are extracted in one tissue. The corresponding P-values in the 2nd comparative tissue are extracted and FDR is performed on this 2nd set of P-values. The number of hits falling below the FDR cutoff (0.05 level = red, 0.01 level = blue) is plotted for each of these windows. This was done for meta-probesets in (A) HObs and (B) LCLs, as well as probesets in (C) HObs and (D) LCLs. (0.32 MB PDF) [file pgen.1000608.s002.pdf]

A

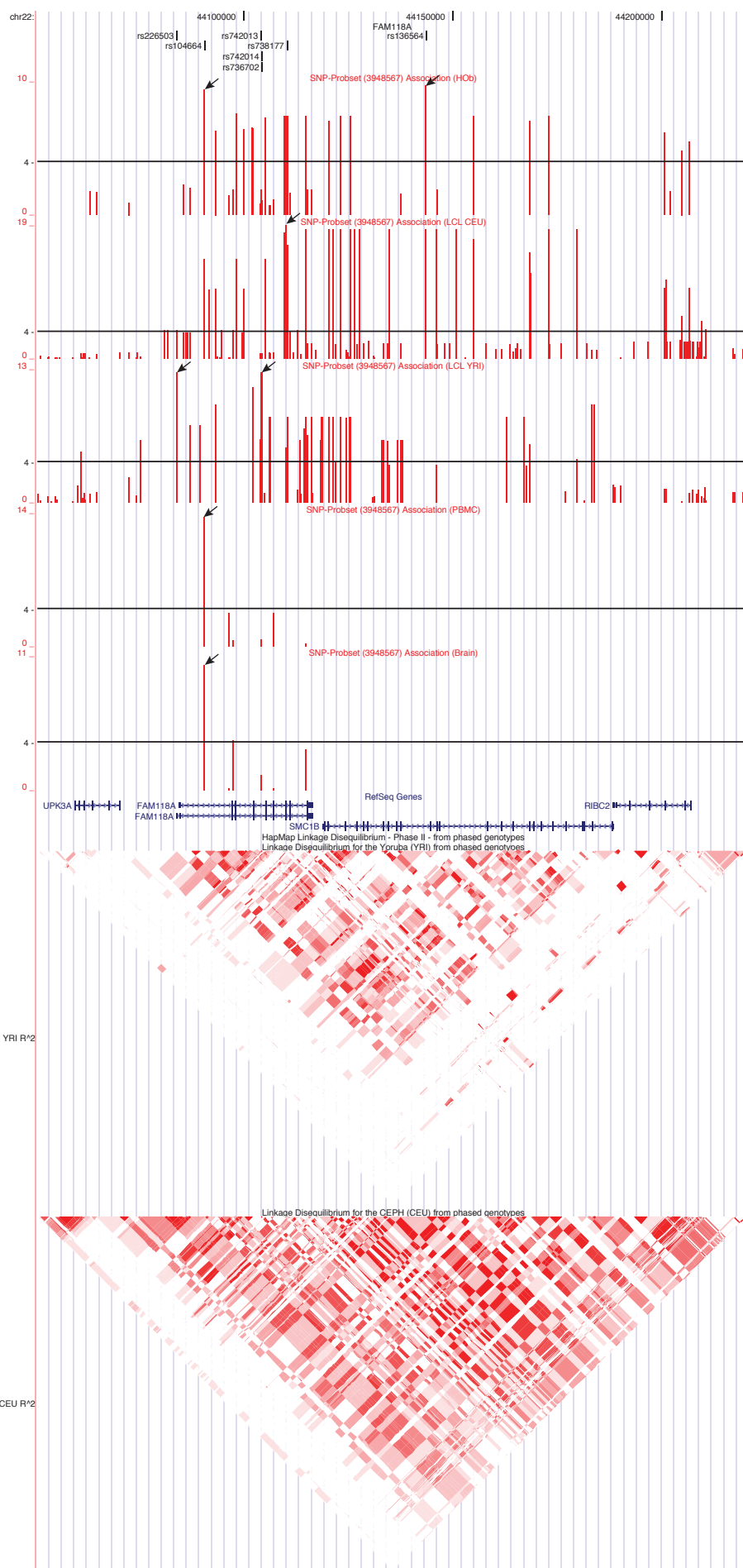

B

C

D

E

F

G

H

I

Supplement: Figure S3 — Tissue-independent cis-associations of FAM118A transcript expression. (A) The top significant SNPs associated with FAM118A transcript expression in different populations and tissues. P-values of association for probeset 3948567 expression scores and SNP genotypes from (B) HOb, (C) HapMap CEU LCL, (D) HapMap YRI LCL (Zhang et al, AJHG 2008), (E) PBMCs (Heinzen et al., PLoS Biology 2008), and (F) Cortical brain tissue (Heinzen et al., PLoS Biology 2008) are shown as vertical bars and represented as -log10PV. (G) RefSeq transcripts in the FAM118A and flanking regions. (H) YRI and (I) CEU linkage disequilibrium blocks. (1.30 MB PDF) [file pgen.1000608.s003.pdf]

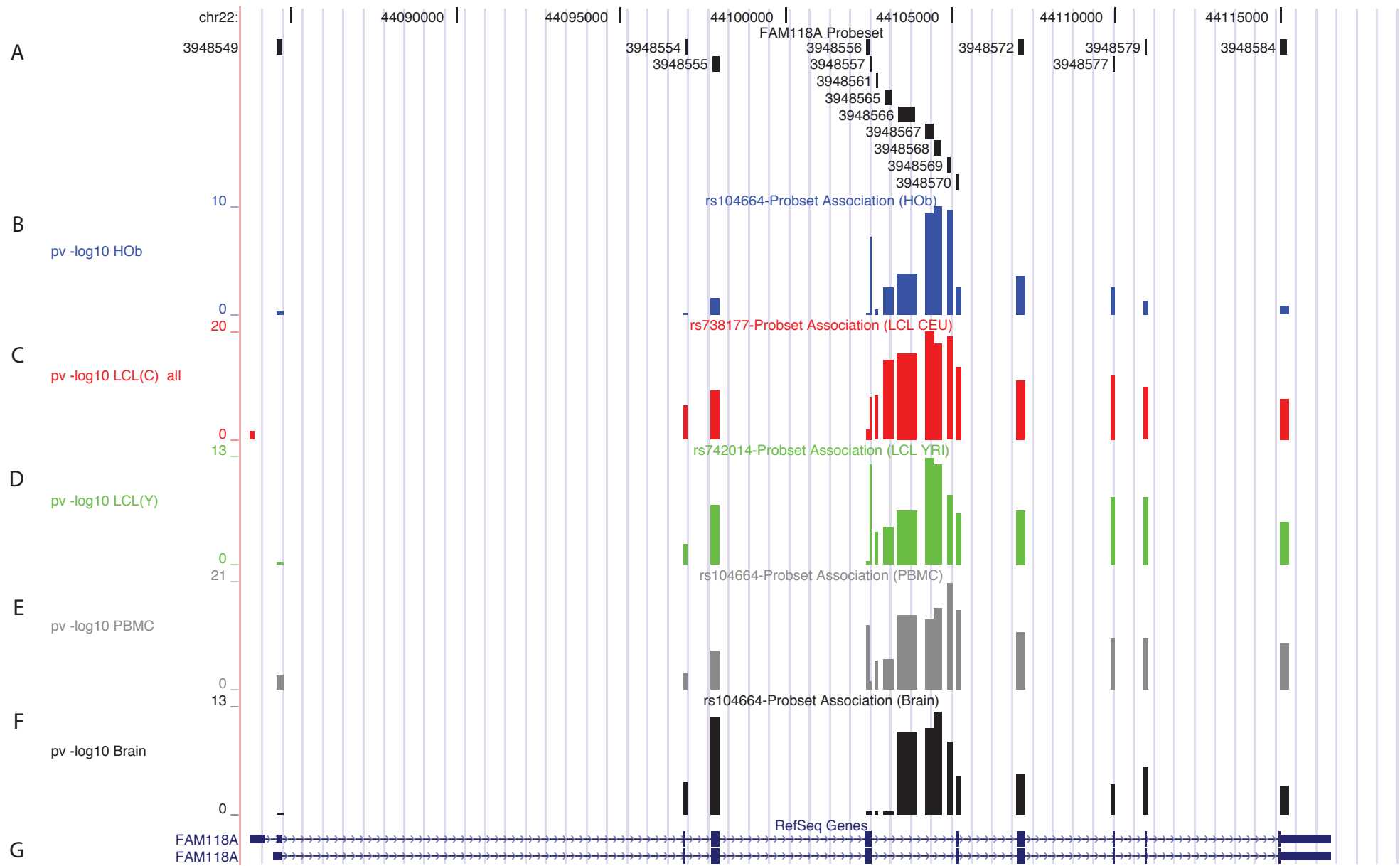

Supplement: Figure S4 — Association of the top significant SNP with all FAM118A probesets in different populations and tissues. (A) All probesets corresponding to the newly identified FAM118A transcript variant. P-values of association for linear regression of the FAM118A probesets in different samples and genotypes of (B) rs104664 in HObs, (C) rs738177 in HapMap CEU LCLs, (D) rs742014 in HapMap YRI LCLs, (E) rs104664 in PBMCs, and (F) Cortical brain tissue are shown as vertical bars and represented as -log10PV. (G) Two different RefSeq transcripts of FAM118A, NM_001104595 and NM_017911. (0.28 MB PDF) [file pgen.1000608.s004.pdf]
